# New antimicrobial peptide kills drug-resistant pathogens without detectable resistance

**DOI:** 10.18632/oncotarget.24582

**Published:** 2018-02-26

**Authors:** Jong-Kook Lee, Tudor Luchian, Yoonkyung Park

**Affiliations:** ^1^ Research Center for Proteinaceous Materials, Chosun University, Gwangju, Korea; ^2^ Department of Physics, Alexandru I. Cuza University, Iasi, Romania; ^3^ Department of Biomedical Science, Chosun University, Gwangju, Korea

**Keywords:** Staphylococcus aureus, clavaspirin peptide (CSP), toll-like receptor-2 (TLR-2), nuclear factor-kappa B (NF-κB), pro-inflammatory cytokine

## Abstract

Clavaspirin peptide (CSP) is derived from the pharyngeal tissues of the tunicate *Styela clava*. The 23-amino acid peptide is histidine-rich and amidated at the N-terminus. CSP possesses low antimicrobial and high hemolytic activity at pH 7.4. Therefore, we designed 4 CSP analogs with substituted hydrophobic amino acids to reduce hydrophobic amino acid interactions. These modifications reduced the aggregation and cytotoxicity of the analogs at pH 7.4. The analogs also showed potent antimicrobial activity by accumulating on bacterial cell surfaces and inducing the lytic mechanism against gram-negative and gram-positive cells at pH 5.5 and 7.4. Moreover, exposure to the CSP-4 analog for up to 29 passages did not induce drug resistance in *Staphylococcus aureus*. Application of CSP-4 to inflamed skin of hairless mice infected with drug-resistant S. *aureus* (DRSA) significantly reduced skin infections without damaging dermal collagen or elastin. Topically applied CSP-4 penetrated 25–40 µm in the dermis within 30 min, reducing the levels of Toll-like receptor-2, nuclear factor kappa B (NF-κB), and the pro-inflammatory cytokines tumor necrosis factor- α (TNF-α) and interleukin-1β (IL-1 β). These results suggest that CSP-4 could be a promising topical antimicrobial agent for skin diseases caused by DRSA such as S. *aureus* CCARM 0027.

## INTRODUCTION

The common use of antibiotics against bacteria has led to the emergence of many antibiotic-resistant pathogen strains. As a result, infections caused by multidrug resistant bacteria have become a significant problem. It is estimated that in Europe alone there are 171,200 healthcare-associated infections with multidrug-resistant *Staphylococcus aureus* (MRSA) each year, affecting both adults and children [1, 2]. Thus, there is an urgent need to develop new antibiotic agents. One potentially effective strategy is to develop new agents derived from antimicrobial peptides (AMPs) [3], which are produced natively as part of the host defense response to microbial infections. Specifically, more than 20 AMPs have been identified in cases of drug-resistant *S. aureus* (DRSA), a leading cause of skin and soft tissue infections. Susceptibility to DRSA-associated infectious disease appears to be related to immunity mechanisms [4–8]. DRSA is associated with porcine reproductive and respiratory syndrome [4], the activation of nuclear factor kappa B (NF-κB) [5]. It is also associated with the production of cytokines, including tumor necrosis factor alpha (TNF-α) and interleukin (IL)-6, IL-1α, and IL-1β [6]; chemokines, including CXCL1, CXCL2, IL-8, and CXCR2 [7]; and AMPs, including defensins and cathelicidin [8].

Marine invertebrates lack acquired or memory-type immunity and rely solely on innate immunity mechanisms [9]. In a previous report, a gene involved in an innate immune mechanism, AMP from marine invertebrates, clavaspirin peptide (CSP), was cloned from a cDNA library prepared from the pharyngeal tissues of a tunicate, *Styela clava*. CSP has a histidine-rich, α-helical structure composed of 23 amino acid residues. It exhibits antimicrobial activity against both gram-negative and gram-positive bacteria at low pH, but its activity is diminished at pH 7.4. In addition, CSP induces hemolysis in both human and bovine red blood cells (RBCs) [10].

Understanding how the toxicity of these AMPs is controlled is very important, as toxicity is one of the major challenges in their general application for treating infection. A leucine, tryptophan, or phenylalanine zipper motif has been reported to play a key role in the toxic activity of AMPs, but does not affect its antimicrobial activity [11–13].

In the present study, we modified CSP by replacing two of the hydrophobic amino acids in its leucine-zipper motif with more hydrophilic residues. The resultant peptide, CSP-4, displayed low cytotoxicity, even at high concentrations, and reduced the spread of bacterial infection in a mouse model of dermal infection. Moreover, CSP-4 exhibited strong pH-independent antimicrobial activity (including against DRSA) stemming from its ability to disrupt the cell membrane of microorganisms. CSP-4 repressed pro-inflammatory cytokines and inhibited DRSA-induced dermatitis in hairless mice. Additionally, CSP-4 did not induce resistance against tested bacteria when compared with commercial antibiotics such as daptomycin and linezolid. Therefore, CSP-4 may have therapeutic potential for dermatitis.

## RESULTS

### Development of New AMPs

**Figure 1:**
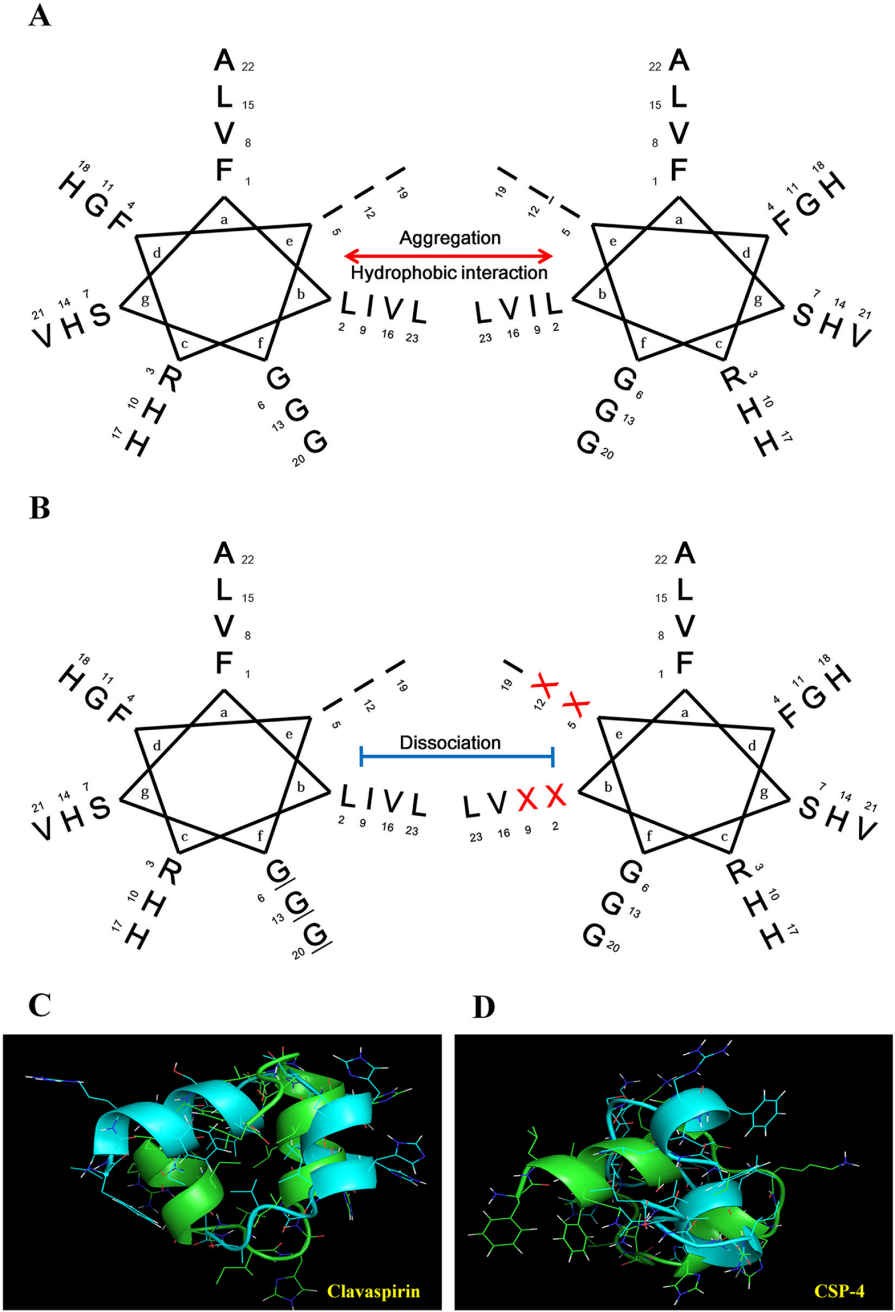
Helical wheel diagram structure of the clavaspirin peptide (CSP) and its analogs (**A**) CSP possesses a 3_10_-helical structure (coiled-coil) and a leucine-zipper motif. The residue positions are labeled from a to g. Hydrophobic interactions between amino acid residues in positions e and b appear in the helical wheel diagram structure. (**B**) Isoleucine and leucine residues at positions 2, 5, 9, and 12 are located in the same face, allowing for the formation of a leucine-zipper. Analogue peptides were designed by substitution of the X position amino acid. We designed 4 peptide analogs by substituting the hydrophobic amino acids (CSP-1, I5A, and I12A; CSP-2, I5K, and I12K; CSP-3, L2K, and I9K; and CSP-4, I9K, and I12K) in the leucine-zipper motif. The resulting analogs were named CSP-1, CSP-2, CSP-3, and CSP-4. Peptide structure analysis was conducted using the PEP-FOLD program (http://bioserv.rpbs.univ-paris-diderot.fr/services/PEP-FOLD/). (**C**) CSP showed peptide-peptide interactions as in a leucine zipper motif. (**D**) CSP-4 structure showed disruption of the repeat leucine and/or isoleucine zipper motif.

Previous studies demonstrated that CSP has weak antimicrobial activity and high hemolytic activity towards mammalian RBCs at physiological pH (pH 7.4) [10]. We therefore designed a set of CSP analogs to produce peptides exhibiting three features essential for therapeutic efficacy against dermatitis infection: 1) minimal aggregation at pH 5.5 and 7.4; 2) strong antimicrobial activity at pH 5.5 and 7.4; and 3) low or no cytotoxicity towards mammalian cells at pH 5.5 and 7.4. CSP has a helix-loop-helix [14] and a leucine-zipper motif (Figure 1A and Figure 1C) [15]. Isoleucine and leucine residues at positions 2, 5, 9, and 12 face each other to form a leucine-zipper motif and contribute to the high hydrophobicity of CSP, leading to its aggregation at pH 7.4. In this study, we prepared four CSP analogs (CSP-1, CSP-2, CSP-3, and CSP-4) by substituting combinations of two of the isoleucine and leucine residues with alanine or lysine to dissociate the leucine and/or isoleucine zipper motif (Figure 1B, Figure 1D and Supplementary Table 1). C_18_ reversed-phase high-performance liquid chromatography confirmed that the hydrophobicity of the synthetic CSP analogs was reduced. Moreover, the analogs exhibited lower minimum inhibitory concentrations (MICs) toward various microorganisms at pH 7.4, but less hemolytic activity and cytotoxicity than CSP against mammalian cells (Supplementary Table 1).

We visualized cell membrane damage induced by the CSP analogues and assessed their binding affinities for human (h)RBCs and HaCaT cells. Treatment with CSP-2 induced membrane shrinkage in hRBCs, whereas rhodamine-labeled CSP-3 bound to the cell membrane of HaCaT cells with high affinity. In contrast, CSP-4 displayed no detectable hemolytic or membrane-binding activity (Supplementary Figure 1). Of the four synthetic analogs, CSP-4 showed the lowest hydrophobicity and cytotoxicity (Table 1 and Supplementary Figure 1). Using a microdilution assay, we compared the antimicrobial activities of CSP-4, melittin, and four conventional antibiotics (erythromycin, ampicillin, gentamicin, and piperacidin) toward *S. aureus* and its drug-resistant strains. As shown in Table 1, the MIC of CSP-4 was lower against drug-resistant *Escherichia coli*, *Pseudomonas aeruginosa*, and *S. aureus* strains than other antibiotic drugs.

**Table 1:** The antibiotic activity of clavaspirin, its analogue peptides and melittin, erythromycin, gentamicin, ampicillin and piperacidin

Microorganism strains	MIC (μM)														
	Cla		CSP-1		CSP-2		CSP-3		CSP-4		Me	Er	Ge	Am	Pi
	I	II	I	II	I	II	I	II	I	II					
**Gram (–)**															
E. coli	2	64	4	8	2	4	2	4	2	4	2	-	-	-	-
*P. aeruginosa*	32	>64	32	32	32	16	32	32	32	32	2	-	-	-	-
L. monocytogenes	16	64	16	64	8	4	16	8	4	4	2	-	-	-	-
**Gram (+)**															
*B. subtilis*	16	64	16	64	4	4	8	16	4	4	2	-	-	-	-
*S. epidermidis*	4	32	4	16	4	32	4	8	4	8	2	-	-	-	-
*S. aureus*	16	64	16	64	8	16	8	8	8	8	2	-	-	-	-
*S. typhimurium*	32	64	32	64	64	4	64	4	64	4	2	-	-	-	-
*P. vulgaris*	8	64	8	64	4	8	4	8	4	8	2	-	-	-	-
**Resistant**															
*E. coli* CCARM*1238*	-	-	-	-	-	-	-	-	-	2	2	-	-	>800	>800
*P. aeruginosa 3543*	-	-	-	-	-	-	-	-	-	4	2	-	-	>800	>800
*P. aeruginosa 3904*	-	-	-	-	-	-	-	-	-	2	2	-	-	>800	>800
*P. aeruginosa 3547*	-	-	-	-	-	-	-	-	-	4	2	-	-	400	200
*P. aeruginosa 4007*	-	-	-	-	-	-	-	-	-	2	2	-	-	>800	>800
*S. aureus* 3126	-	-	-	-	-	-	-	-	-	8	2	128	32	-	-
*S. aureus* 0027	-	-	-	-	-	-	-	-	-	8	2	>256	64	-	-
*S. aureus* 5157	-	-	-	-	-	-	-	-	-	8	2	16	16	-	-
*S. aureus* 1635	-	-	-	-	-	-	-	-	-	8	2	>256	>256	-	-
*S. aureus* 4761	-	-	-	-	-	-	-	-	-	8	4	>256	32	-	-
*S. aureus* 5159	-	-	-	-	-	-	-	-	-	8	8	16	16	-	-
*S. aureus* 3359	-	-	-	-	-	-	-	-	-	8	4	>256	64	-	-
*S. aureus* 2122	-	-	-	-	-	-	-	-	-	8	4	>256	128	-	-
*S. aureus* 1630	-	-	-	-	-	-	-	-	-	8	4	>256	64	-	-
*S. aureus* 1870	-	-	-	-	-	-	-	-	-	8	4	>256	16	-	-
*S. aureus* 3511	-	-	-	-	-	-	-	-	-	8	4	>256	>256	-	-
*S. aureus* 5156	-	-	-	-	-	-	-	-	-	8	4	>256	>256	-	-
*S. aureus* 3518	-	-	-	-	-	-	-	-	-	8	4	>256	>256	-	-

Minimum inhibitory concentrations (MIC) were determined in 10 mM sodium phosphate buffer (I, pH 5.5; and II, pH 7.4) containing 10% Luria broth (LB). Drug-resistant *Pseudomonas aeruginosa* and *Staphylococcus aureus* bacteria were isolated from hospitalized otitis media patients (Chonnam National University’s hospital, Korea). This strain (with CCARM number) was obtained from the Culture Collection of Antimicrobial Resistant Microbes in Korea. Cla, Me, Er, Ge, AM and Pi are Clavaspirin, Melittin, erythromycin, gentamicin, ampicillin and piperacidin, respectively. (–) is not determined.

### Induction of resistance

**Figure 2:**
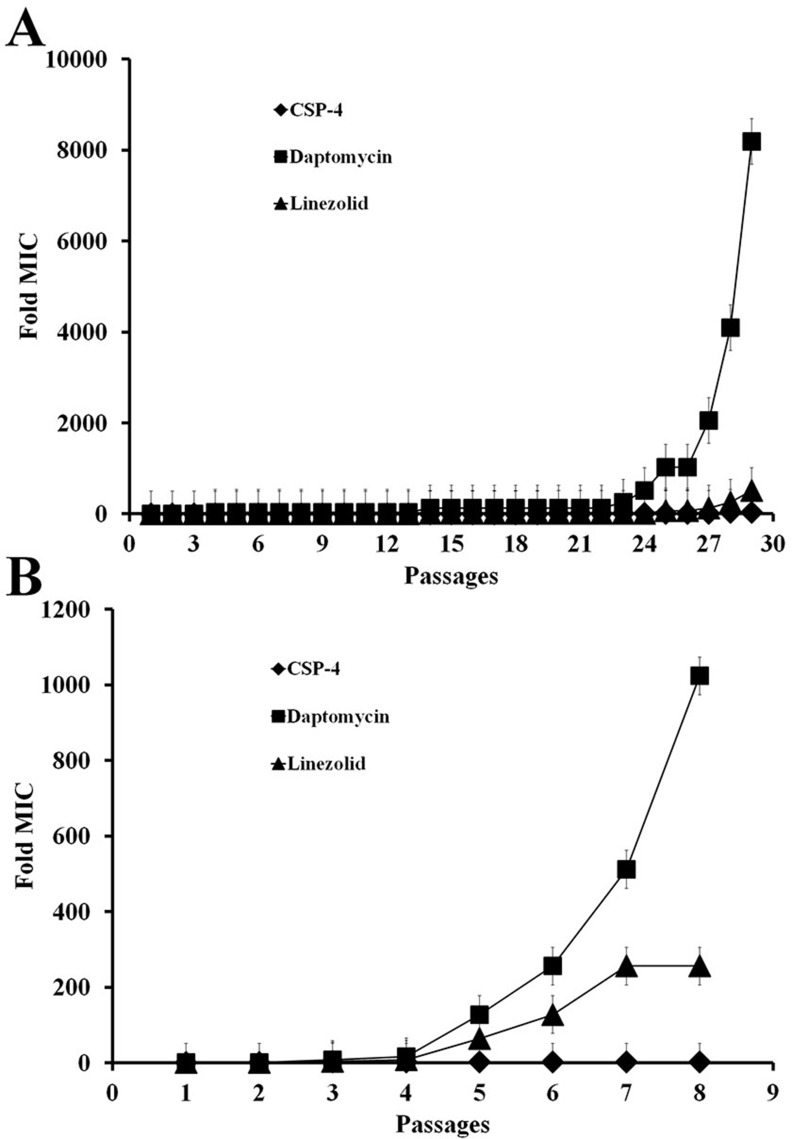
CSP and its analogues on the bacterial cell membrane do not lead to antibiotic resistance (**A**, **B**) Development of resistance in *S. aureus* CCARM 29213 cells exposed to CSP-4, daptomycin, or linezolid at pH 5.5 and pH 7.4. The Y-axis shows the relative change in MIC from the first passage. All values represent the mean **±** SD of three individual experiments (*p* < 0.05, one-way ANOVA).

To determine the MIC of CSP-4 against *S. aureus*, we cultured a single colony of *S. aureus* ATCC 25923 and then passaged the cells up to 30 times to determine whether prolonged exposure to CSP-4 led to drug resistance. The conventional antibiotics linezolid and daptomycin were also tested as positive controls. The MIC for linezolid was increased by approximately 200× after 8 passages at pH 7.4, whereas the MIC for daptomycin was increased by approximately 1000× after 8 passages at pH 7.4, and over 8000× after 29 passages at pH 5.5 (Figure 2A, 2B). In contrast, the MIC for CSP-4 increased by only approximately 2× (16 vs. 8 μM) after exposure for 29 passages. Thus, CSP-4 exhibited long-term antibacterial activity against *S. aureus* with minimal development of drug resistance.

### Anti-inflammatory effect

**Figure 3:**
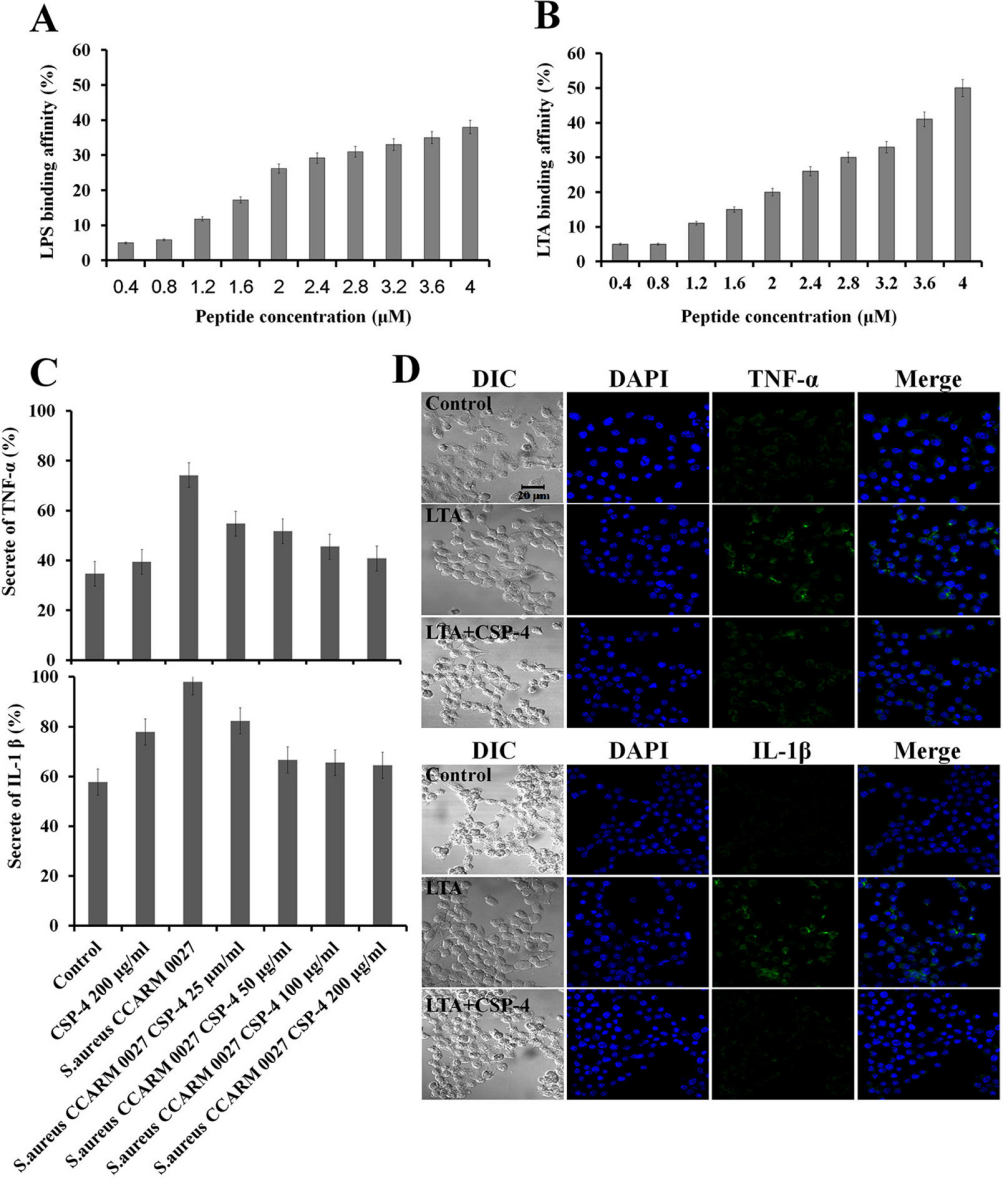
Binding of CSP-4 to LPS and LTA and CSP-4-induced inhibition of pro-inflammatory cytokines in RAW 264.7 macrophages (**A**, **B**) CSP-4 binding at the indicated concentrations to *E. coli* LPS in 5 mM HEPES buffer, as measured using 2.5 μM dansyl polymyxin B (*Ex*. 340 nm, *Em*. 485 nm). All values represent the mean **±** SD of three individual experiments (*p* < 0.05, one-way ANOVA). (**C**) Secretion of TNF-α (top) and IL-1β (bottom) from RAW 264.7 cells exposed to *S. aureus* CCARM 0027 (1 × 10^6^ cfu/mL) and subsequently treated with CSP-4 (25, 50, 100, and 200 μg/mL). All values represent the mean **±** SD of three individual experiments (*p* < 0.05, one-way ANOVA). (**D**) The effect of CSP-4 (100 µg/mL) on *S. aureus* LTA-induced expression of TNF-α and IL-1β in RAW 264.7 macrophages. The cells were stimulated with LTA (1 µg/mL) for 3 h in the presence and/or absence of CSP-4 at pH 5.5 (top) or pH 7.4 (bottom), fixed, permeabilized, and stained with antibodies. Scale bar, 20 μm.

We assessed the binding affinity of CSP-4 for LPS and LTA. LPS or LTA (9 µg) was incubated with dansyl polymyxin B (2.5 μM) in 5 mM HEPES buffer and the concentration of CSP-4 was increased in 0.4-μM increments. Addition of CSP-4 led to a dose-dependent increase in fluorescence intensity, indicating successful binding of CSP-4 to LPS or LTA (Figure 3A and Figure 3B). CSP-4 also inhibited the expression of the pro-inflammatory cytokines TNF-α and IL-1β in RAW 264.7 macrophages exposed to *S. aureus* CCARM 0027. As shown in Figure 3C, CSP-4 significantly decreased TNF-α and IL-1β levels compared to in cells exposed to *S. aureus* CCARM 0027 alone. The ability of 100 μg/mL CSP-4 to neutralize 1 μg/mL lipopolysaccharide (LPS) prompted us to test whether CSP-4 suppressed the inflammatory response elicited by LPS or lipoteichoic acid (LTA). CSP-4 similarly reduced TNF-α and IL-1β levels in RAW 264.7 cells treated with 1 μg/mL *S. aureus* LTA (Figure 3D). In the absence of CSP-4, TNF-α and IL-1β were predominantly localized to the cytosol, but were absent from cells treated with 100 µg/mL CSP-4. Thus, CSP-4 not only exerts antimicrobial effects, but also inhibits the production of pro-inflammatory cytokines during *S. aureus* CCARM 0027 infection.

### *In vivo* efficacy

**Figure 4:**
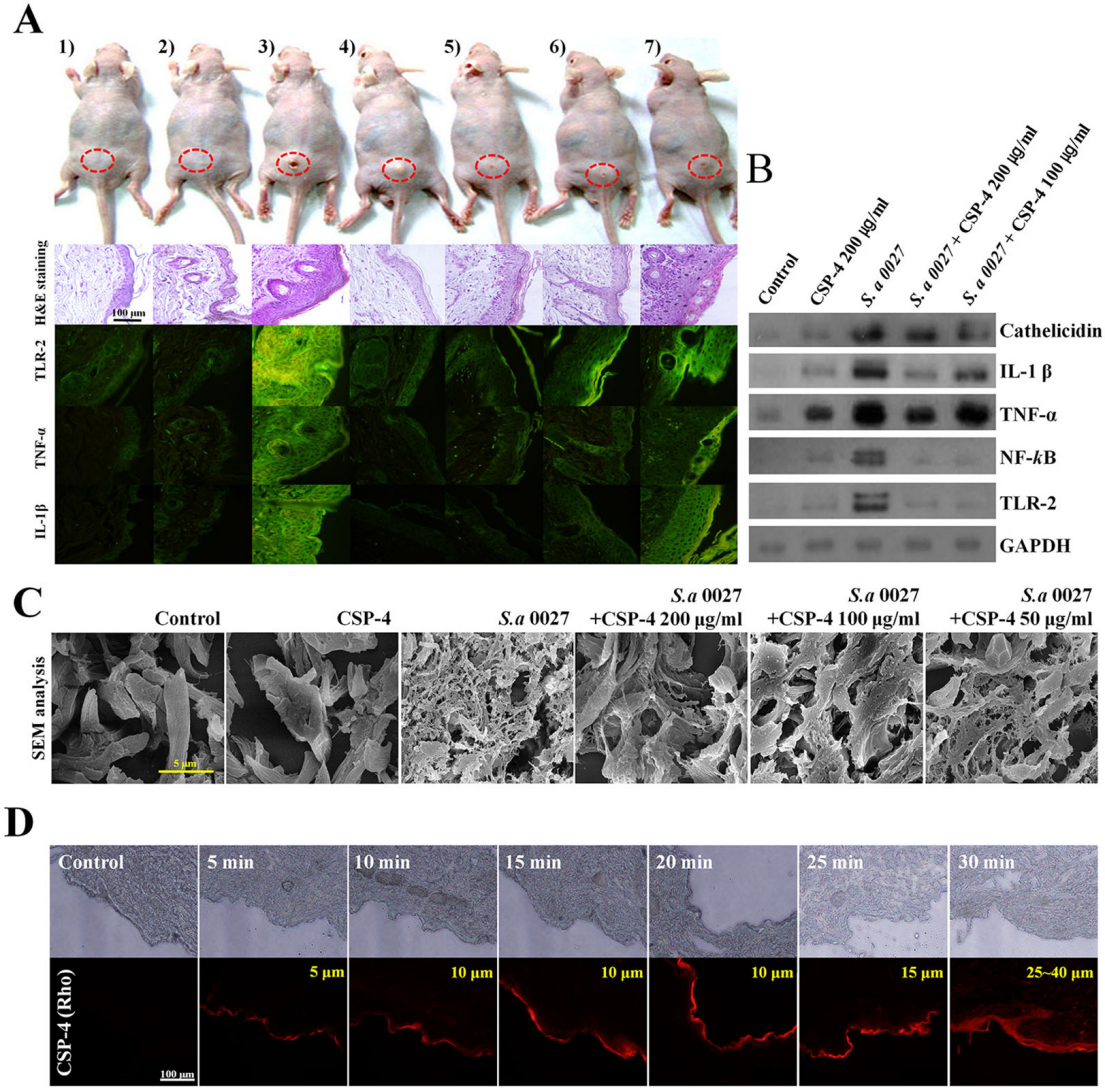
Application of CSP-4 to hairless mouse skin (**A**) Hairless mouse skin infected with *S. aureus* CCARM 0027 (1 × 10^8^ cfu/mL) leading to dermatitis. Mice were treated topically with 200, 100, 50, or 25 µg/mL CSP-4 and were sacrificed 7 days later. Dorsal skin tissue was collected and stained using hematoxylin and eosin or FITC-conjugated anti-TLR-2, anti-TNF-α and anti-IL-1β antibodies, as indicated. From left to right, samples were treated with 1) PBS, 2) 200 µg/mL CSP-4, 3) *S. aureus* CCARM 0027 at 1 × 10^8^ cfu/mL, 4) *S. aureus* and 200 µg/mL CSP-4, 5) *S. aureus* and 100 µg/mL CSP-4, 6) *S. aureus* and 50 µg/mL CSP-4, and 7) *S. aureus* and 25 µg/mL CSP-4. Scale bar, 100 μm. (**B**) Western blots showing the effect of CSP-4 on *S. aureus* CCARM 0027-induced expression of the indicated pro-inflammatory signaling proteins and AMP cathelicidin. (**C**) Scanning electron micrographs showing morphological changes in the dermis of the hairless mouse under the indicated conditions. *S. aureus* CCARM 0027 was added at 1 × 10^8^ cfu/mL. Scale bar, 5 μm. (**D**) Time-dependent penetration of hairless mouse skin by rhodamine-labeled CSP-4 (red; 200 μg/mL). Scale bars, 100 µm. Numbers in the upper right of the fluorescence micrographs indicate depth of penetration.

We used *S. aureus* CCARM 0027-infected hairless mice as a model of dermal infection and inflammation. *S. aureus* CCARM 0027 induced skin inflammation and caused histological changes (Figure 4A). Hematoxylin and eosin-stained sections showed that the epidermis of infected mice was thicker than that of PBS-treated controls or mice inoculated with 200 µg/mL CSP-4 alone. Moreover, immune cells infiltrated the epidermis of *S. aureus* CCARM 0027-infected mice. CSP-4 (at 200 µg/mL and 100 µg/mL) elicited a dose-dependent decrease in epidermal thickness and immune cell infiltration. Immunohistochemical analysis with fluorescein isothiocyanate (FITC)-labeled anti-TNF-α, anti-IL-1β, and anti-TLR-2 antibodies revealed that skin samples from mice infected with *S. aureus* CCARM 0027 without CSP-4 contained significantly higher levels of pro-inflammatory cytokines and TLR-2 than infected mice treated with 100 µg/mL or 200 µg/mL CSP-4. Western blot analysis showed that the *S. aureus* membranes contained LTA, which is thought to bind to TLR-2 on immune cells such as macrophages, activating innate immunity in response to gram-positive pathogen recognition. This, in turn, activated NF-κB signaling and induced TNF-α, IL-1β, and cathelicidin production to form a line of defense against infectious microorganisms (Figure 4B). However, mice treated with 200 μg/mL or 100 μg/mL CSP-4 showed concentration-dependent decreases in TLR-2, NF-κB, TNF-α, IL-1β, and cathelicidin levels. Examination of dermal collagen and elastin using scanning electron microscopy (SEM) showed that in skin sections from mice treated with PBS or CSP-4 alone, the dermal collagen and elastin were not damaged, whereas the skin of *S. aureus* CCARM 0027-infected mice showed substantial collagen and elastin damage that was dose-dependently reduced by CSP-4 (Figure 4C). This data suggests that the skin of *S. aureus* CCARM 0027-infected hairless mice undergoes morphological and immunohistochemical changes indicated by inflammation, and that CSP-4 greatly attenuates that response.

### Distribution of topically applied CSP-4 on the skin

CSP-4 will be applied to the skin topically from induced dermatitis against *S. aureus* CCARM 0027. Using fluorescence microscopy with rhodamine-labeled CSP-4, we observed that the topically applied peptide permeated into deep layers of the skin. Fluorescence was detected at skin depths of 25–40 μm. The fluorescent signal was detected throughout the epidermis within approximately 15 min and then throughout the dermis within 30 min. This result demonstrates that topically applied CSP-4 is rapidly distributed deep into infected skin (Figure 4D and Supplementary Figure 2).

### Mechanism of action

**Figure 5:**
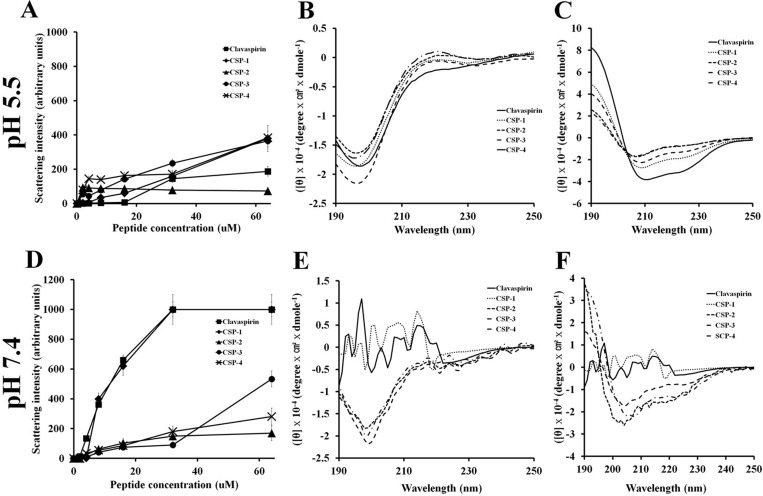
Aggregation of clavaspirin and its analogue peptides at pH 5.5 and pH 7.4 Aggregation of clavaspirin and its analogue peptides. (**A**, **B**) Peptides incubated for 12 h at room temperature in 10 mM sodium phosphate buffer at pH 5.5 or pH 7.4 were monitored for structural changes using light scattering on an LS55 luminescence spectrometer (excitation 400 nm and emission 400 nm). Circular dichroism (CD) spectra analyses of peptides were incubated in 10 mM sodium phosphate (**D**, **E** left panel) or 30 mM SDS (**C**, **F** right panel). The secondary structures of CSP and its analogue peptides were determined using Far-UV CD spectra. All values represent the mean **±** SD of three individual experiments (*p* < 0.05, one-way ANOVA).

We used light scattering to analyze the self-aggregation of CSP and its analogues (Figure 5). Although CSP and CSP-1 aggregated in a dose-dependent manner at pH 7.4, CSP-4 showed very little self-aggregation at pH 5.5 or pH 7.4 (Figure 5A and Figure 5D). Given that CSP-4 is less hydrophobic than other CSP analogues, we suggest that the increased antimicrobial activity and reduced cytotoxicity were achieved by replacing hydrophobic residues with more hydrophilic residues (Supplementary Table 1). To investigate the structure-activity relationship of the peptides, we used circular dichroism (CD) spectroscopy to determine their secondary structures in aqueous solution (10 mM sodium phosphate at pH 5.5 and pH 7.4) and a membrane-mimetic environment (30 mM SDS, 1% w/v, at pH 5.5 and pH 7.4). CSP and its analogs showed random coiled structures in aqueous solution at pH 5.5 or pH 7.4 (Figure 5B and Figure 5E), but all peptides formed α-helical structures in the membrane-mimetic environment (Figure 5C and Figure 5F). However, CSP and CSP-1 also showed an unordered structure between 220 and 190 nm in both the aqueous and membrane-mimetic environments at pH 7.4, indicating aggregation (Figure 5E).

**Figure 6:**
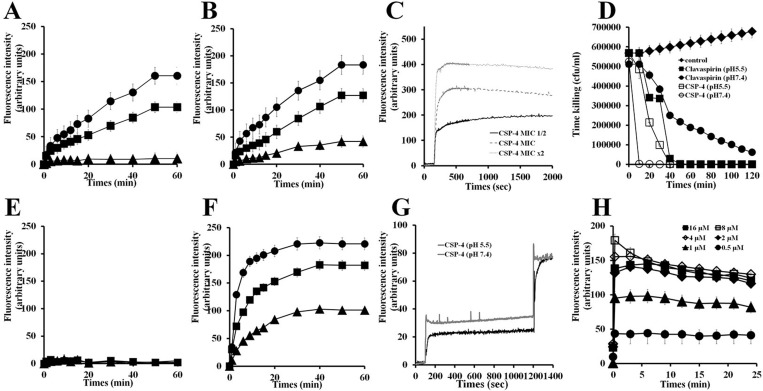
Mechanism of action of CSP and its analogue peptides against *E. coli* and *S. aureus* *E. coli* (2 × 10^7^ cfu/mL) were incubated with 1 μM SYTOX-Green for 15 min. CSP (**A**, **E**) and CSP-4 (**B**, **F**) were added when basal fluorescence reached a constant value in 10 mM sodium phosphate buffer at pH 5.5 (A, B) or pH 7.4 (E, F). (**C**) Fluorescence was monitored at the indicated times (*Ex*. 485 nm and *Em.* 520 nm). Hyperpolarization was induced by incubation for 5 min on ice followed by incubation at 37° C with disC_3_-5 (0.5 mM) for 10 min. (**D**) CSP-4 killing kinetics. *E. coli* cells in not a growth phase were adjusted to OD_600_ 0.06, CSP-4 was then added at its MIC or 2× MIC, and changes in fluorescence were monitored continuously (*Ex*. 622 nm and *Em*. 670 nm) and plotted as arbitrary units. (**G**) CSP-4-induced release of FD-FITC-dextran from LUV liposomes (PE/PG, 7/3, w/w). Effect of pH on CSP-4-induced release of FD-FITC-dextran from LUV liposomes (PE/PG, 7/3, w/w). CSP-4 was added at the MIC to LUVs entrapping FD-FITC-dextran (100 mg/mL) dye in HEPES buffer at pH 5.5 or pH 7.4. The dotted line shows the simultaneous determinations of bacterial viability after exposure of *E. coli* cell to the MIC or 2× MIC of CSP-4. (**H**) Effect of CSP-4 on *S. aureus* membrane permeability. *S. aureus* cells (2 × 10^7^ cfu/mL) were incubated for 15 min with 1 μM SYTOX-Green. CSP-4 was added when basal fluorescence reached a constant value in 10 mM sodium phosphate buffer. The increase in fluorescence was monitored at the indicated times (*Ex*. 485 nm and *Em*. 520 nm). All values represent the mean **±** SD of three individual experiments (*p* < 0.05, one-way ANOVA).

Cationic SYTOX-Green dye does not penetrate the cell membrane unless the membrane is damaged, and its fluorescence intensity increases upon binding to intracellular nucleic acids [16]. Using SYTOX-Green to assess peptide-induced membrane disruption in *E. coli* and *S. aureus* (Figure 6), we found that treating bacteria with CSP-4 elicited a dose-dependent increase in fluorescence intensity, indicating rapid disruption of the bacteria cell membrane at pH 5.5 (Figure 6A and Figure 6B) and pH 7.4 (Figure 6E and Figure 6F). In contrast, CSP showed little ability to disrupt bacterial membranes at pH 7.4. Measurement of DisC3–5 fluorescence confirmed the dose-dependent disruption and resultant depolarization of the *E. coli* membrane by CSP-4 (Figure 6C).

The killing kinetics for CSP and CSP-4 against *E. coli* at pH 5.5 and pH 7.4 indicated that CSP-4 reduced bacterial growth faster than CSP at their respective MICs (Figure 6D). The time-dependent depolarization of FD-FITC-dextran-loaded large unilamellar vesicles (LUVs) suggests that cells treated with CSP-4 were more depolarized at pH 7.4 than at pH 5.5 (Figure 6G). The CSP-4 peptide is dose-defendant increased of membrane disruption on *S. aureus* membrane (Figure 6H).

**Figure 7:**
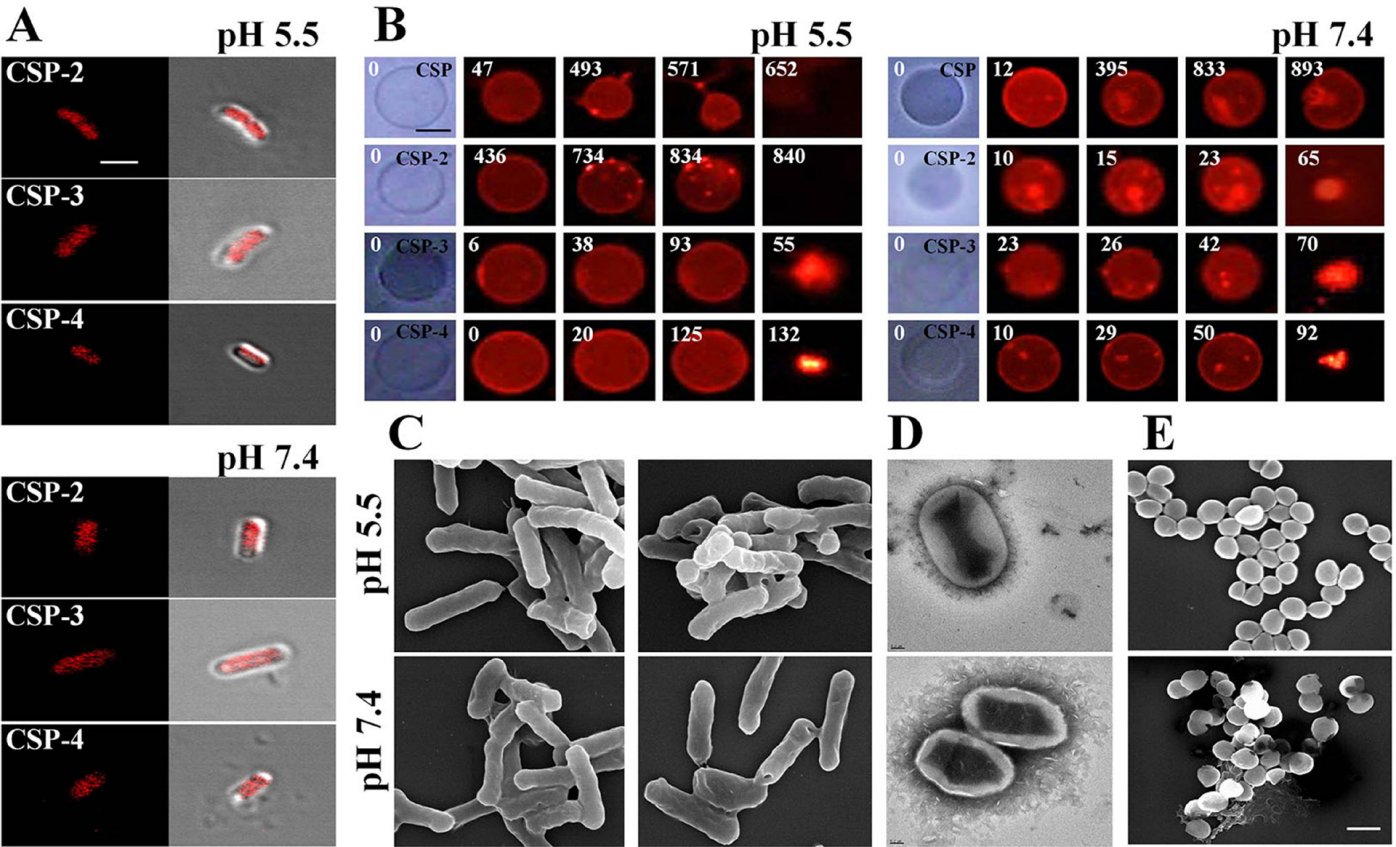
Accumulation of CSP and its analogues on the bacterial cell membrane (**A**) Localization of the indicated CSP analogues on *E. coli*. Cells were incubated for 20 min with the MICs of rhodamine-labeled AMPs. Cells were visualized using confocal laser scanning microscopy. Scale bar, 5 μm. (**B**) Effect of CSP (20 μM; pH 5.5 or pH 7.4) and the indicated CSP analogues on GUVs (PE/PG/PE-rhodamine). Scale bar, 50 μm. Scanning (**C**, **E**) and transmission (**D**) electron micrographs show disruption of the *E. coli* and *S. aureus* cell membrane by CSP-4 at 1/2 MIC in 10 mM sodium phosphate buffer (pH 5.5 and pH 7.4). The panels show formation of large pores after exposure to CSP-4. *S. aureus* cells were cultured in the absence (top) and presence (bottom) of CSP-4. Scale bar in C and E, 5 µm; scale bar in d, 2 nm.

We investigated the mechanism of action of the CSP analogs using confocal laser scanning microscopy with tetramethylrhodamine-labeled peptides at their MICs. Tetramethylrhodamine-labeled CSP-2, CSP-3, and CSP-4 were observed on *E. coli* cell surfaces at pH 5.5 and pH 7.4 (Figure 7A). Electron microscopic examination revealed that after binding to the membrane, CSP-4 at its MIC induced membrane damage in both *E. coli* and *S. aureus* cells at pH 5.5 and pH 7.4 (Figure 7C–7E). The lytic activities of the CSP analogues were further examined using giant unilamellar vesicles (GUVs) composed of PE/PG/PE-rhodamine (69/30/1), which were visualized and recorded using a CCD camera. Treatment with 20 μM CSP induced lysis of the GUVs at pH 5.5, but not at pH 7.4. In contrast, 20 μM concentrations of all CSP analogs induced lysis of the GUV membrane at both pH 5.5 and pH 7.4. Moreover, analogues in which an isoleucine residue was substituted with lysine disrupted the GUV membrane more quickly at pH 5.5 than at pH 7.4 compared to those substituted with alanine (Figure 7B and Supplementary Figure 3). This data confirms that the CSP-4 peptide acts on the *S. aureus* membrane via a carpet-like mechanism, in which CSP-4 quickly interacts with the inner membrane through its electrostatic interaction with LTA.

## DISCUSSION

Infectious diseases caused by various microorganisms, including antibiotic-resistant bacteria, affect a considerably large human population. In particular, dermatitis infections such as those caused by S. aureus are a serious problem in hospitals. These types of infections have increased because of antibiotic resistance. *S. aureus* has been reported to cause serious infections of the skin and soft tissue [17] and is highly invasive, causing life-threatening complications, including bacteremia, pneumonia, abscesses of various organs, meningitis, osteomyelitis, endocarditis, and sepsis [18, 19]. These infections are often nosocomial, resulting from the widespread emergence of DRSA and MRSA, and are an important public health concern [2]. Thus, alternatives to conventional antibiotic drugs are urgently needed [20–22]. Previous studies of AMPs have focused on their isolation, characterization, potential mechanisms of action, and effects of amino acid substitution. Therefore, AMPs have also been studied *in vivo*, on tissues infected by antibiotic-resistant bacteria. In this study, we report a newly designed CSP-4 peptide, effective in a bacterial dermatitis mouse model infected with DRSA, non-toxic, and able to suppress the effect of various pro-inflammatory cytokines and chemokines *in vivo*.

CSP is an AMP with a helix-loop-helix structure that is rich in hydrophobic amino acids. CSP has leucine and isoleucine residues at positions 2, 5, 9, and 12, corresponding to the leucine and/or isoleucine zipper motif (N-terminal amino acid), which contains three isoleucine residues and one leucine residue located adjacently. In other reports, AMPs with leucine or phenylalanine zipper motifs showed high cytotoxicity [11–13, 23–26]. In the current study, we analyzed the peptide structure using the PEP-FOLD program (http://bioserv.rpbs.univ-paris-diderot.fr/services/PEP-FOLD/). CSP showed peptide-peptide interactions (N-terminal region) driven by the leucine zipper motif (Figure 1A and Figure 1C). Therefore, to reduce hydrophobicity, leucine and/or isoleucine in the zipper motif (N-terminal region) were substituted with alanine or lysine residues; these substituted CSPs showed little or no cytotoxicity and maintained activity. CSP-4 was developed by substituting lysine for the isoleucine residues at positions 9 and 12. The CSP-4 structure disrupts the repeat leucine and/or isoleucine zipper motif at the N-terminal (Figure 1B and Figure 1D). This structure is a key determinant of the hemolytic and cytotoxic properties of the peptide, as CSP-4 exhibited potent antimicrobial activity against both drug-susceptible reference strains and multidrug-resistant bacterial strains (Table 1) while showing remarkably low cytotoxicity in hRBCs and mammalian cells (Supplementary Table 1 and Supplementary Figure 1). This result was observed because of the low hydrophobic peptide-peptide interaction or aggregation by substitution of isoleucine to lysine (Figure 1B, Figure 1D and Figure 5). CSP, CSP-1, and CSP-3 peptides induced higher self-aggregation, resulting from hydrophobic interactions (induced by the leucine zipper motif), at pH 5.5 and pH 7.4, but not the CSP-2 and CSP-4 peptides. CSP induced low aggregation at pH 5.5 (Figure 5A); however, aggregation increased at pH 7.4 (CSP, CSP-1 and CSP-3) (Figure 5D). The CSP-2 and CSP-4 analogue were not affected (Figure 5A and Figure 5D) by varying the pH. Additionally, we analyzed the changes in secondary structure using CD spectra. CSP showed a helical structure in SDS buffer (Figure 5C), but not in 10 mM sodium phosphate buffer at pH 5.5 (Figure 5F). However, CSP showed high aggregation in SDS buffer and 10 mM sodium phosphate buffer at pH 7.4 (Figure 5E). CSP-2, CSP-3, and CSP-4 formed stable helical structures within/without 30 mM SDS at pH 5.5 and pH 7.4 (Figure 5C and Figure 5F). CSP-4, with its dissociated leucine zipper motif, showed strong antimicrobial activity against DRSA and no or low cytotoxicity in hRBC and HaCaT cells, independent or regardless of pH (Table 1, Supplementary Table 1, and Supplementary Figure 1).

An increasing number of studies have reported the emergence of daptomycin or linezolid-nonsusceptible *S. aureus* in patients during daptomycin therapy. *S. aureus* is an important human pathogen responsible for numerous infections. Therefore, we used either CSP-4, daptomycin, or linezolid to treat *S. aureus* infections. We observed that, following prolonged exposure, the MIC of daptomycin against *S. aureus* increased by as much as 8000-fold, and that of linezolid was also greatly increased (Figure 2). Therefore, we concluded that these bacterial cells had developed resistance to both daptomycin and linezolid over a relatively short period of time, but not to CSP-4 at pH 5.5 (Figure 2A) and pH 7.4 (Figure 2B). The resistance to daptomycin (0.25 μM MIC) and linezolid (4 μM MIC) was induced throughout 24 passages under the same conditions at pH 5.5 (Figure 2A), while at pH 7.4, daptomycin (4 μM MIC) and linezolid (8 μM MIC) induced resistance after only 5 passages (Figure 2B). This finding suggests that bacterial cells do not develop resistance to CSP-4 as rapidly as they do to conventional antibiotics (such as daptomycin and linezolid).

The outer membrane of gram-positive bacteria contains LTA, which is secreted during cell division and is recognized by TLR-2 on immune cells via interactions with LTA-binding protein (Figure 3B). Upon LTA recognition (e.g., in diseases such as colonization and infection of the skin), the NF-κB signaling pathway is activated, leading to the expression of various pro-inflammatory cytokines (TNF-α and IL-1β). We found that the levels of signaling proteins (TLR2 and NF-κB) and pro-inflammatory cytokines (TNF-α and IL-1β) in skin tissue were lower following treatment with 100 µg/mL or 200 µg/mL CSP-4 than in untreated mice. CSP-4 appeared to neutralize LTA on the membrane of DRSA cells, down-regulating the expression of pro-inflammatory cytokines (Figure 3C, Figure 4A and Figure 4B). In addition, the outer membrane of gram-negative bacteria contains LPS, which is secreted during cell division and is recognized by TLR-4 on immune cells *via* interactions with LPS-binding proteins (Figure 3A). A similar effect was caused by CSP-4 when RAW 264.7 macrophages were treated with 1 µg/mL *S. aureus* LTA (Figure 3D). Thus, CSP-4 appears to have both antimicrobial and anti-inflammatory effects.

We examined the *in vivo* effects of CSP-4 using a mouse model of bacterial dermatitis. CSP-4 reduced the number of FITC-conjugated anti-TLR-2, anti-TNF-α, and anti-IL-1β antibodies in the skin of nude mice, indicating that the peptide achieved good penetration of the skin, enabling it to exert its bactericidal effect (Figure 4A). In addition, seven days after dermal infection with DRSA CCARM 0027, the epidermis and dermis of otherwise untreated hairless mice were swollen and damaged, reflecting infiltration by macrophages and disruption of dermal collagen and elastin. In mice that also received CSP-4, however, skin damage was substantially reduced, suggesting that CSP-4 suppresses inflammatory responses *in vivo* (Figure 4C).

TLRs recognize specific patterns of pathogen components of bacteria, viruses, fungi, and parasites. The cell wall of gram-positive bacteria contains LTA as an important pro-inflammatory constituent, which stimulates immune cells by activating TLR-2 [27]. This signaling occurs through the myeloid differentiation primary response gene 88-dependent pathway, which leads to the downstream activation of NF-kB and mitogen-associated protein kinase signaling pathways (ERK-CREB, JNK-AP-1, and p38). This signaling is responsible for the induction of pro-inflammatory cytokines, chemokines, AMPs, and adhesion molecules [28]. Previous reports have shown that keratinocytes as well as macrophages and other immune cells in the epidermis and dermis secrete AMP cathelicidin during infections, and that bacteriostatic or bactericidal activities towards pathogens such as *S. aureus* involve α- and β-defensins, RNase7, and dermicidin [29–33]. In the present study, *S. aureus* CCARM 0027 infection led to significant increases in cathelicidin levels, but the increase was attenuated by 200 µg/mL or 100 µg/mL CSP-4, again demonstrating its capacity to reduce inflammation (Figure 4B).

Recently, the HIV-TAT PTD-coupled RP-1 fusion protein was shown to penetrate the dermis when applied to the surface of rat skin [24]. We hypothesized that CSP-4 could be induced to similarly distribute into the epidermis and dermis if applied to the skin surface. We found that the peptide was predominantly localized in the epidermis after 5 min, but after 15 min CSP-4 had begun to penetrate the dermis, and substantial distribution into the dermis was observed within 30 min (Figure 4D). The topical use of peptides has been widely studied because of its importance for treating skin diseases, and there is growing interest in using peptide application as a topical vaccination to treat skin conditions. For instance, Rothbard *et al.* [34] were the first to report that direct delivery of peptide R7 (polyarginine-7) into the skin leads to therapeutically effective and dose-dependent drug distribution, showing the presence of activity both *in vitro* and *in vivo* [34].

Additionally, we confirmed the mode of action of CSP-4 in bacteria. The hydrophobicity of the designed peptides, analyzed based on retention time using C_18_ reversed-phase high-performance liquid chromatography, was significantly reduced. The peptide analogs showed a significant reduction in aggregation at pH 7.4, whereas CSP aggregated in a concentration-dependent manner (Figure 5D). Because hemolysis and cytotoxicity were assayed at pH 7.4, these data indicate that peptide aggregation contributes to cytotoxicity at that pH. The hydrophobicity of aggregated peptides was markedly increased. This aggregation was observed in the CD spectra at pH 7.4 [35] (Figure 5E). CSP-4 showed potent antimicrobial activity and reduced cytotoxicity in HaCaT and hRBC cells (Table 1 and Supplementary Table 1). The protein displayed a random coil structure and its α-helical structure was induced in 30 mM SDS buffer at pH 5.5 and pH 7.4 (Figure 5). CSP-4 possesses four histidine residues and has an increased cationic charge at pH 5.5 because of histidine’s pKa of 6.03 [36, 37]. Although its high cationicity allows CSP-4 to rapidly bind to anionic bacterial membranes through electrostatic interactions (such as LPS and LTA), this strong binding prevents easy permeation into the lipid bilayer. The leucine zipper motif-containing CSP was modified into a more hydrophilic peptide by substituting lysine for isoleucine residues (Figure 1). As a result, the CSP-4 analog not only showed reduced cytotoxicity because of disaggregation, but also showed enhanced antibacterial activity at pH 7.4 compared to CSP (Table 1 and Supplementary Table 1).

In the present study, measurements of SYTOX-Green uptake, membrane depolarization, kill kinetics, and FITC leakage all indicated that CSP-4 exerted a rapid membranolytic effect. The difference in action of CSP-4 between pH 5.5 and pH 7.4 was purely kinetic, suggesting that peptide binding to the cell membrane is pH-dependent, reflecting its cationicity. The high cationicity of CSP-4 enabled its rapid binding to the anionic components of the bacterial membrane (LTA and LPS) through electrostatic interactions. After binding to the outer membrane *via* LPS in *E. coli* and LTA in *S. aureus* (Figure 3), CSP-4 quickly induced cell lysis (Figure 6H and Figure 7E).

**Figure 8:**
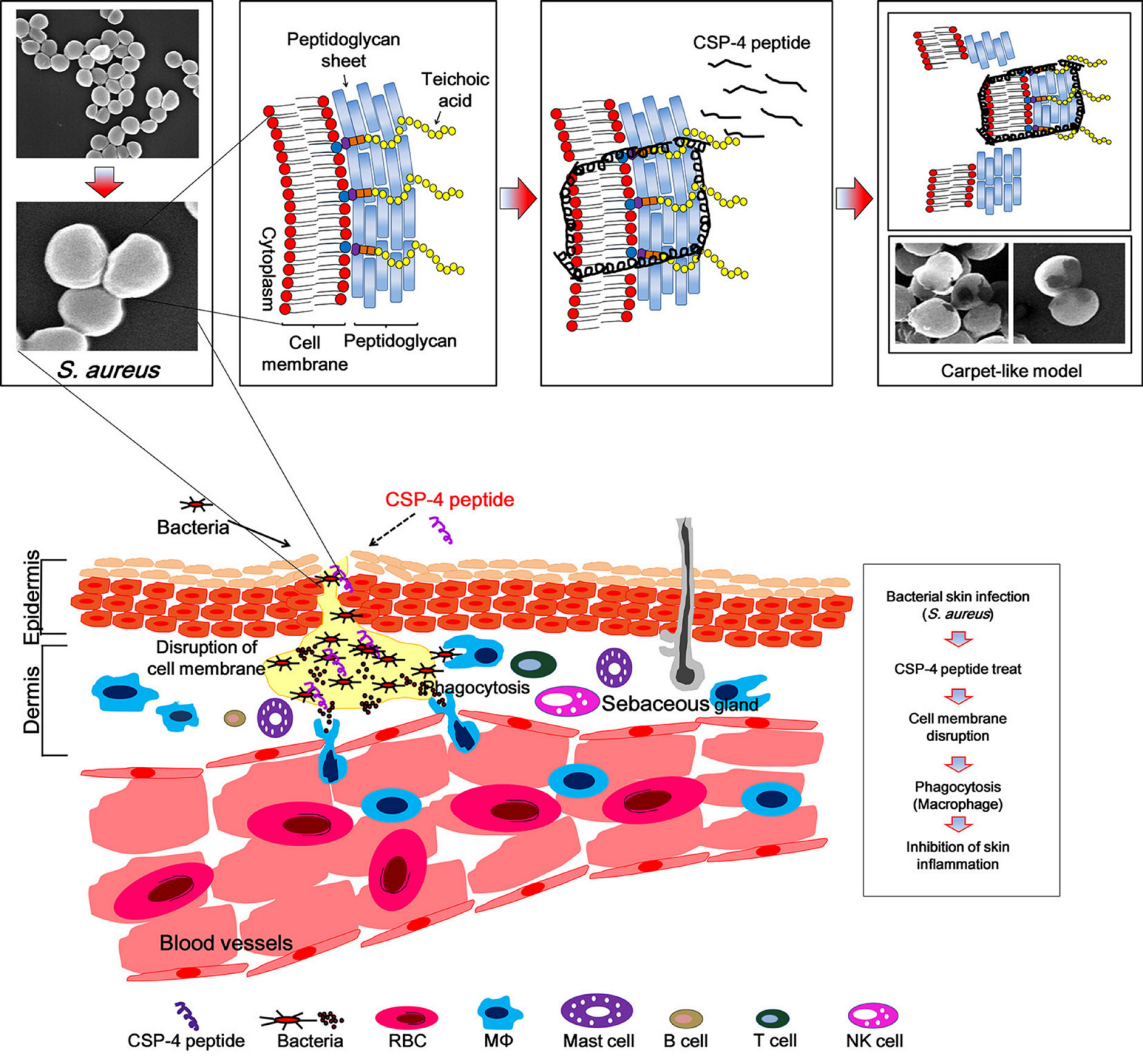
Schematic diagram of the proposed mechanism of action of CSP-4 based on a Toll-like receptor-mediated immune response against DRSA CSP-4 rapidly disrupts the *S. aureus* cell membrane using a carpet-like mechanism, whereby CSP-4 quickly interacts with the inner membrane through its electrostatic interaction with LTA. CSP-4 interacted with *S. aureus* membranes contain LTA, which binds to TLR-2 on immune cells such as macrophages. This activates the NF-κB signaling pathway and induces the production of cathelicidin and other peptides that combat microorganisms.

We are suggesting that during CSP-4 peptide treatment, the pus formed on the skin will exhibit a rapid action mechanism on the bacterial membrane surface of *S. aureus*. Additionally, CSP-4 may exhibit a stronger bactericidal activity against *S. aureus* induced dermatitis when it is accumulated in the hair follicles on the skin [38]. In these conditions, the peptide initially accumulates on the bacterial membrane and an early electrostatic interaction occurs between its lysine and/or histidine amino acids and LTA. Subsequently, the peptide undergoes a hydrophobic interaction with the lipid head groups to permeabilize the *S. aureus* membrane, perhaps through a carpet-like mechanism [39]. Our SEM analysis supports this interpretation (Figure 8). Furthermore, we confirmed significantly reduced skin damage in CSP-4–treated (compare with untreated peptide) samples, which induced inflammation on the hairless mouse skin from *S. aureus* CCARM 0027. The skin represents a formidable barrier to the delivery of small- and large-molecule therapeutic agents. Hair follicles and sweat glands represent potentially efficient penetration pathways for topically applied substances. Our findings suggest that CSP-4 is a potential therapeutic AMP that can be applied topically onto skins undergoing infections caused by a wide range of MRSA.

## MATERIALS AND METHODS

### Peptide synthesis, microorganisms, and antimicrobial activity

The method for peptide synthesis has been described in detail previously [40]. *E. coli* ATCC 25922 (LB media), *P. aeruginosa* ATCC 15692 (NB+0.5% NaCl media), *Listeria monocytogenes* ATCC 3710 (LB media), *Staphylococcus epidermidis* ATCC 12228 (LB media), and *S. aureus* ATCC 29213 (TSB media) were obtained from the American Type Culture Collection (Manassas, VA, USA). *Bacillus subtilis* KCTC 1998 (LB media), *Salmonella typhimurium* KCTC 1926 (NB media), and *Proteus vulgaris* KCTC 2433 (NB media) were obtained from the Korean Collection for Type Cultures (Jeollabuk-do, Korea). *E. coli* CCARM 1238 was received from the Culture Collection of Antibiotic Resistant Microbes at Seoul Women’s University, Korea. Drug-resistant *P. aeruginosa* 3543, 3904, 3547, and 4007 and *S. aureus* 3126, 0027, 5157, 1635, 4761, 5159, 3359, 2122, 1630, 1870, 3511, 5156, and 3518 were resistant strains isolated from patients with otitis media in a Chonnam National University’s hospital.

The antibacterial activities of AMPs (melittin, clavaspirin, and its analogs) and conventional antibiotic drugs (erythromycin, gentamicin, ampicillin, and piperacidin) were determined using microdilution assays. Bacterial cells were cultured at 37° C in appropriate culture media at pH 5.5 or pH 7.4 until collection during the mid-log phase and suspended in 10 mM sodium phosphate buffer (pH 5.5 or pH 7.4) supplemented with 10% culture medium. Thereafter, 2× serial dilutions of each peptide (0.25–128 μM) or antibiotic (0.25–256 μM) were added to sterile 96-well plates, and aliquots of cell suspension (5 × 10^5^ colony forming units (cfu)/mL) were seeded into each well. The samples were then incubated at 37° C for 24 h. At the end of the incubation, the minimum inhibitory concentrations (MICs) of the peptides were determined using a VERSA Max microplate reader (Molecular Devices Co., Sunnyvale, CA, USA) at a wavelength of 600 nm. The lowest peptide concentration that completely inhibited growth was defined as the MIC. MIC values were calculated as the mean of 3 independent experiments conducted in triplicate [40, 41].

### Hemolysis

Fresh human red blood cells (hRBCs) from healthy donors were centrifuged at 800 × *g* and washed with phosphate-buffered saline (PBS) until the supernatant was clear. Twofold serial dilutions of the peptides in PBS were added to a 96-well plate, after which the hRBCs were added to obtain a final concentration of 8% (vol/vol). The samples were then incubated with mild agitation for 1 h at 37° C. The samples were then centrifuged at 800 × *g* for 10 min, and the absorbance of the supernatant was measured at 414 nm. All the measurements were performed in triplicate, and the percentages of hemolysis were calculated using equation 1:

$$\%\;\text{hemolysis}=\left[\left(A_{414}\;\text{in peptide solution}-A_{414}\;\text{in}\;\text{PBS}\right)/\left(A_{414}\;\text{in}\;0.1\%\text{Triton X}-100-A_{414}\;\text{in}\;\text{PBS}\right)\right]\times 100\;$$ (1)

Where 100% hemolysis was defined as the absorbance measured from hRBCs exposed to 1% Triton X-100 and zero hemolysis was characterized using hRBCs alone in PBS [40].

### Cytotoxicity

To examine the cytotoxic effects of the peptides, HaCaT (human keratinocyte) cells were cultured in Dulbecco’s modified Eagle’s medium (DMEM) supplemented with antibiotics (100 U/ml penicillin and 100 μg/ml streptomycin) and 10% fetal calf serum at 37° C in a humidified chamber under a 5% CO_2_ atmosphere. Growth inhibition was evaluated using 3-(4, 5-dimethylthizol-2-yl)-2, 5-diphenyltetrazolium bromide (MTT) assays to measure cell viability. After cells were seeded into a 96-well plate at a density of 2 × 10^4^/well and incubated for 24 h, twofold serial dilutions of each peptide in DMEM were added to the wells, and the cells were incubated for an additional 24 h at 37° C. Thereafter, 10 μl of MTT (5 mg/ml) was added to each well, and the plate was incubated for 4 h. The supernatants were then removed, and 50 μl of DMSO was added to each well to dissolve any remaining precipitate. Finally, the absorbance at 570 nm was measured using a microtiter reader [40].

### Resistance development assay

To determine the MICs of CSP-4, a single colony of *S. aureus* ATCC 29213 was cultured at 37° C in tryptic soy broth, as well as daptomycin and linezolid, which served as commercial antibiotic positive controls. The final concentrations of CSP-4 ranged from 8 to 256 μM at pH 5.5 and from 8 to 16 μM at pH 7.4; daptomycin ranged from 0.25 to 2048 μM at pH 5.5 and from 4 to 4096 μM at pH 7.4; linezolid ranged from 4 to 4096 μM at pH 5.5 and from 8 to 2048 μM at pH 7.4. For each agent, the MIC was determined daily for 29 days at pH 5.5 and/or for 8 days at pH 7.4 using cells from wells containing half the MIC (1/2 MIC well). Briefly, cells from a 1/2 MIC well were suspended in an appropriate culture medium and incubated for 24 h at 37° C, after which the suspensions were adjusted to 5 × 10^5^ CFU/mL in 10 mM sodium phosphate buffer (pH 5.5 or 7.4) with 10% tryptic soy broth, and mixed with agents in the same concentration ranges. All MICs were determined in duplicate [42].

### Peptide-LTA and LPS binding affinity assay

Lipoteichoic acid (LTA) and lipopolysaccharide (LPS) binding affinity assays were performed using a Perkin-Elmer LS55 fluorometer (Waltham, MA, USA). *S. aureus* LTA and *E. coli* LPS (9 µg) were incubated with 2.5 μM dansyl-polymyxin B in 5 mM HEPES buffer for 5–10 min at pH 7.4. The CSP-4 peptide was added in 0.4 μM increments from 0.4 to 4 μM. The increase in fluorescence intensity was assessed using a spectrofluorometer at an excitation wavelength of 340 nm and an emission wavelength of 482 nm (Perkin-Elmer LS55) [43].

### Cell culture and protein extraction

To examine cytokine expression elicited by *S. aureus* CCARM 0027, RAW 264.7 mouse macrophages were cultured in DMEM supplemented with antibiotics (100 U/μM penicillin and 100 μg/mL streptomycin) and 10% fetal calf serum in 12-well plates at 37° C in a humidified chamber with an atmosphere containing 5% CO_2_. Expression of the pro-inflammatory cytokines TNFα and IL-β was evaluated after the addition of *S. aureus* CCARM 0027 (1 × 10^6^ cfu/mL) for 30 min at 100° C. After treatment, the RAW 264.7 cells (1 × 10^6^ cells/mL) were incubated for an additional 24 h with or without CSP-4 (25, 50, 100, and 200 µg/mL). The cells were then collected and the protein was extracted using PRO-PREP^™^ protein extraction solution (iNtRON Biotechnology, Gyeonggi-do, Korea).

### Measurement of TNF-α and IL-1β

RAW 264.7 cells (1 × 10^6^ cells/mL) were seeded into a 12-well plate and incubated for 24 h as described above. Untreated cells (negative control) and cells exposed to *S. aureus* CCARM 0027 (1 × 10^6^ cfu/mL) were incubated for 12 h in the absence or presence of CSP-4 (25, 50, 100, and 200 µg/mL) in DMEM supplemented with 10% bovine serum. The levels of pro-inflammatory cytokines in the supernatants were determined after 12 h using mouse anti-TNF-α and anti-IL-1β antibodies based on absorbance at 450 nm using a microplate reader (VERSA Max microplate reader, Molecular Devices). Cytokine concentrations were also determined using TNF-α and IL-1β enzyme-linked immunosorbent assay kits (Koma Biotech, Seoul, Korea) [44].

### Immunocytochemistry

RAW 264.7 cells (1 × 10^6^ cells/mL) were cultured for 12 h as described above and incubated for 6 h in the presence of *S. aureus* LTA (1 μg/mL) with or without CSP-4 (100 μg/mL). The cells were then washed in PBS, fixed in 4% paraformaldehyde (15 min), permeabilized using 0.5% Triton-X 100 diluted in PBS, washed again 3 times by gently shaking in PBS for 5 min each, blocked in a blocking buffer (5% bovine serum albumin in PBS), and then added to an 8-well plate (200 μL/well), in which they were incubated for 1 h with gentle shaking. Primary antibodies (TNF-α, AbFrontier, Seoul, Korea, AB1793; and IL-1β AbFrontier, AB1413; 1:50 dilution) were then added to the cells for 24 h at 4° C, after which the cells were washed 3 times in PBS (5 min/wash). A secondary antibody was applied (goat anti-mouse IgG-HRP, LF-SA 5001-conjugated; 1:200 dilution) for 1 h at 37° C, and nuclei were stained using DAPI (0.25 µg/mL). The staining was visualized under a confocal laser-scanning microscope (LSM510; Carl Zeiss; Gottingen, Germany) [45].

### Immunohistochemistry

Male hairless mouse (aged 6 weeks, 5 mouse/group, total 7 groups) were injected intraepidermal with *S. aureus* CCARM 0027 (1 × 10^8^ cfu/ml). The CSP-4 peptide (200, 100, 50, 25 μg/ml) was injected intraeptidermal into mice at 1 h after the S. *aureus* CCARM 0027 injection, samples of inflamed skin was collected after 7 days and 4-μm-thick sections were prepared as described in the preceding section. Each sample was then incubated for 30 min at 24° C with 5% bovine serum albumin, mouse anti-Toll-like receptor-2 (TLR-2) (AbFrontier, AB24192), mouse monoclonal anti-TNF-α (AbFrontier, AB1793), and mouse polyclonal anti-IL-1β (AbFrontier, AB1413). The samples were then washed with Tris-buffered saline containing Tween 20 buffer and incubated with HRP LF-SA5001-conjugated goat anti-mouse IgG, and stained with hematoxylin and eosin. The stained sections were examined under a fluorescence microscope [46].

### Topical assay

Topical delivery of rhodamine-labeled CSP-4 was evaluated on hairless mouse skin. Male hairless mice (6 weeks old) bred by Hoshino Laboratory Animals, Inc. (Bandou-shi, Japan) were purchased from Japan SLC, Inc. (Hamamatsu, Japan). After anesthetizing 5 mice per group (3 groups) with isoflurane, rhodamine-labeled CSP-4 (200 μg/mL) was spread on the hairless mouse skin. The skin was then examined for reactions every 5 min for 30 min. Skin tissue was collected and washed once in PBS, fixed in 4% paraformaldehyde for 12 h, dehydrated in 50% and 100% ethanol (2 h each), and washed 3 times in xylene substitute (1 h/wash). Paraffin-embedded samples were cut into 4 μm-thick sections (Microtome; Thermo Scientific, Waltham, MA, USA) and examined under a fluorescence microscope (IX71; Olympus; Tokyo, Japan) [38].

### Western blot analysis

Western blot analysis was performed as using changes in protein expression. Briefly, proteins extracted from the hairless mouse skin were separated for 3 h using 15% SDS-polyacrylamide gel electrophoresis and transferred to polyvinylidene fluoride membranes (Bio-Rad, Hercules, CA, USA) for 1 h at 90 V. The membranes were then incubated overnight at 4° C in 5% skim milk containing anti-GAPDH (LF-PA0018; Santa Cruz Biotechnology, Santa Cruz, CA, USA), mouse anti-TLR2 (AbFrontier, AB24192), mouse anti-NF-κB, (Santa Cruz Biotechnology, SC-71675), mouse anti-TNF-α (AbFrontier, AB1793), mouse anti-IL-1β (AbFrontier, AB1413), and mouse anti-cathelicidin (AbFrontier, AB93357). The membranes were then washed in Tris-buffered saline containing Tween 20 and incubated with the horseradish peroxidase (HRP)-conjugated secondary antibodies LF-SA5002 goat anti-rabbit IgG and LF-SA5001 goat anti-mouse IgG. The blots were developed using a Western Blot Detection kit (AbFrontier, LF-QC0103) [45].

### Aggregation assay

Light scattering assays were performed with clavaspirin and its analogue peptides using a Perkin-Elmer LS55 fluorometer. Peptides were dissolved in 10 mM sodium phosphate buffer (pH 5.5 or pH 7.4) and incubated for 12 h at 37° C. Light scattering was excited at 400 nm and the emission was scanned at 400 nm [35].

### CD analysis

Circular dichroism (CD) spectra were recorded at 25° C on a Jasco 810 spectropolarimeter (Jasco, Oklahoma City, OK, USA) equipped with a temperature control unit. A 0.1-cm path length quartz cell was used with 50 μM peptide solutions under various conditions, including 10 mM sodium phosphate and 30 mM SDS (w/v) at pH 5.5 and pH 7.4. At minimum, 5 scans were acquired and averaged to reduce the signal-to-noise ratio from 250 to 190 nm. Mean residue ellipticity ([θ], degcm^2^dmol^-1^) was calculated using eq. 2:

[θ]=θ obs/10ιc (eq.2)

where θ obs is the measured signal (ellipticity) in millidegrees, ι is the optical path-length of the cell in cm, and c is the concentration of peptide in M and is calculated as the mean residue molar concentration (number of constructed residues of peptide × molar concentration of peptide (M)) [40].

### SYTOX-green uptake analysis

*S. aureus* CCARM 259237 cells were grown to the mid-log phase and suspended in 10 mM sodium phosphate buffer supplemented with 10% culture medium at pH 7.4 and 37° C, and then washed and suspended (2 × 10^7^ cfu/mL) in 10 mM sodium phosphate buffer (pH 7.4) and incubated with 1 μM SYTOX-Green for 20 min in the dark. After the addition of peptides at the appropriate concentrations (1/2 MIC, MIC, 2× MIC, and/or 4× MIC), time-dependent increases in fluorescence, which is caused by the binding of the cationic dye to intracellular nucleic acids, was monitored using a Perkin-Elmer LS55 fluorometer (excitation wavelength, 485 nm; emission wavelength, 520 nm) [40].

### Membrane depolarization of intact bacterial cells

The membrane depolarizing activities of the peptides were evaluated in *E. coli* cells using the membrane potential-sensitive fluorescent dye DiSC3-5 (3,3′-dipropylthiadicarbocyanine iodide). Two batches of bacterial cells (experiment and control) were grown to mid-log phase at 37° C, harvested, and washed three times with buffer A (20 mM glucose, 5 mM HEPES [pH 7.2]). The cells were then resuspended to an *A*600 of 0.05 in buffer A containing 0.1 M KCl, after which DiSC3-5 was added to a final concentration of 0.1 M and the mixture was incubated for 60 min until the fluorescence level stabilized. The peptides were then added to bacterial suspensions, and the changes in fluorescence were continuously recorded (excitation at 622 nm and emission at 670 nm) for 30 min [36].

### Membrane disruption analysis

Large unilamellar vesicles (LUVs) were prepared using the freeze-thaw method. The desired mixture of phospholipids (PE/PG, 7:3) was dissolved in chloroform, dried in a glass tube under nitrogen, and then lyophilized overnight to remove solvent residues. The dry phospholipid films were resuspended in 1–2 mL of 5 mM HEPES buffer (pH 5.5 or pH 7.4) containing 100 μg/mL FD-FITC-dextran, and the resulting mixtures were vortexed. LUVs were prepared using eight freeze-thaw cycles in liquid nitrogen and incubated in a water bath at 50° C. Once vesicles were prepared, suspensions of LUVs were extruded 10 times through polycarbonate membranes with 0.2-μm diameter pores using an Avanti Mini-Extruder (Avanti polar Lipids Inc., Alabaster, AL, USA), after which vesicles with entrapped FD-FITC-dextran were separated from free FD-FITC-dextran by gel filtration chromatography with a Sephadex G-50 column. The vesicle concentration was determined using standard phosphate assays. LUVs were entrapped of FD-FITC dextran in suspension giving 10 μM phospholipid (PE/PG, 7:3). And then, the liposome was incubated with MIC of CSP-4 in HEPES buffer (pH 5.5 or pH 7.4). The fluorescence of the released FD-FITC-dextran was assessed using a spectrofluorometer (Perkin-Elmer LS55) at an excitation wavelength of 490 nm and emission wavelength of 520 nm. Complete (100%) release was achieved by addition of Triton X-100 to a final concentration of 0.1% (w/v) [47].

### Time-kill kinetics

*E. coli* were grown overnight in LB broth (pH 5.5 or pH 7.4) and diluted to an OD_600_ of 0.05. CSP-4 was then added so that the final concentration was either the MIC or 1/2 MIC. *E. coli* cells were then sampled at 1-min intervals for the first 10 min and at 10-min intervals thereafter for 2 h. The cultures were then diluted 50-fold in PBS buffer and plated on LB agar. Colonies were counted after 24 h [48].

### Confocal laser scanning microscopy analysis

The cellular distributions of clavaspirin and its analogues were determined in *E. coli* cells using confocal laser scanning microscopy and rhodamine-labeled peptides. The cells were incubated for 24 h at 37° C in LB media (pH 5.5 or pH 7.4). Rhodamine-labeled peptides were added to 100 μL of cells at the respective MICs. After incubation for 20 min, the cells were pelleted by centrifugation at 4000 × *g* for 5 min and washed three times with ice-cold 10 mM sodium phosphate buffer (pH 5.5 or pH 7.4). Rhodamine-labeled peptides were imaged using a LSM 510 Laser Scanning microscope (Carl Zeiss). The cells were illuminated using a 405-nm line diode laser and 543-nm helium neon laser. The light was directed through a UV 148/154/633 beam splitter, and images were recorded digitally in a 512 × 512 pixel format [40].

### Preparation and visualization of GUVs

Giant unilamellar vesicles (GUVs) were prepared using the electroformation method. Briefly, phospholipid mixtures were prepared in chloroform-methanol (9:1). The mixtures used were PE/PG/PE-rhodamine (69/30/1) and PE/PG (7:3). One hundred microliters of lipid mixture were then deposited onto indium tin oxide (ITO)-coated glass slides (25 × 35 × 1.1 mm, Sigma-Aldrich, St. Louis, MO, USA), which were then spin-coated at 600 rpm for 5 min. After removing residual organic solvents by evacuation in a vacuum for at least 2 h, two ITO glass slides (one with a lipid film) were arranged with a poly(dimethylsiloxane) spacer to form an electroformation chamber (25 × 25 × 1 mm). The chamber was filled with 5 mM HEPES buffer (pH 5.5 or pH 7.4) containing 0.1 M sucrose through a hole in the spacer. A 1.7 V (peak to peak), 10 Hz AC field was then immediately applied to the ITO slides using a function generator (Agilent 33220A, Agilent Technologies, Santa Clara, CA, USA). After 1.5 h, the electric field was changed to 4 V, 4 Hz for 10 min to detach the liposomes formed on the slides. The liposome solution was gently removed from the electroformation chamber, and aliquots were diluted in 5 mM HEPES buffer containing 0.1 M glucose. Aliquots of the resultant GUV suspension were deposited on microscope slides and allowed to settle for 1 min. The settling occurred because of the density difference between the sugar solutions inside and outside the liposomes. The liposomes were then examined under an inverted fluorescence phase contrast microscope (IX71, Olympus), and images were recorded using a digital CCD camera (DP71, Olympus) and video recorder. Images were analyzed using software provided by the manufacturer [49].

### SEM analysis

For scanning electron microscopy (SEM), *S. aureus* cells were cultured in LB medium (pH 5.5 or pH 7.4), washed 3 times with 10 mM sodium phosphate buffer, and stirred at 4000 rpm. CSP-4 was added to the cells and incubated at 25° C. After incubation, the cells were fixed in 4% glutaraldehyde for 20 min and dehydrated in 50% and then 100% ethanol (10 min each step) at 37° C. In addition, 7 days after inducing inflammation in hairless mouse skin tissue by infection with drug-resistant *S. aureus* CCARM 0027 strain or *E. coli* bacteria, samples of skin tissue were isolated, washed in PBS, fixed in 4% paraformaldehyde overnight at 4° C, and dehydrated in 50% and then 100% ethanol (2 h each step) at 37° C. Samples were then gold-coated and visualized by SEM (JSM-7100F; JEOL, Tokyo, Japan) [50].

### TEM analysis

For transmission electron microscopy (TEM), *E. coli* cells were cultured in LB medium (pH 5.5 or 7.4), and then washed 3 times with 10 mM sodium phosphate buffer (pH 5.5 or pH 7.4) at 4000 rpm. CSP-4 was added to the cells at 25° C. After incubation, the cells were applied to glow-discharged carbon-coated copper grids for 1 min. The grids were then rinsed in saline buffer and stained with 2% (w/v) uranyl acetate, after which electron micrographs were recorded using a FEI Technei 12 microscope (FEI Company, Hillsboro, OR, USA) with an acceleration voltage of 120 kV [40].

### Statistics

The data were analyzed using SPSS version 20.0 (Chicago, IL, USA). Values are presented as means ± SD. One-way ANOVA was used to compare resistance development, LPS or LTA binding affinity, TNF-α, IL-1β, peptide aggregation, SYTOX-Green uptake, and time-kill kinetics levels between the absence and/or presence of CSP-4. A *P*-value less than 0.05 were considered statistically significant.

### Study approval

This study was approved by the Institutional Ethics Committee of Chosun University, and all healthy donors provided printed informed consent before the start of experimentation. All procedures complied with the ethical standards of this committee as well as the checklist for ethical concern of cytotoxicity studies. Human red blood cells (RBCs) were obtained from newly collected blood samples from 5 healthy donors at the Chosun University Hospital in Kwangju, Republic of Korea. Furthermore, we obtained an ethics agreement from the Institutional Ethics Committee of Chosun University. The authors of this article were unsighted to all individual information from the donors, who remained nameless throughout the experimental process. After collection, blood samples were directly stored at 4° C until required. The mouse studies were performed in accordance with the National Institutes of Health guidelines for the ethical treatment of animals. All animal processes also conformed to the Experimental Animal Center of Chosun University guidelines. All work was covered by the following license from the Experimental Animal Center of Chosun University: CIACUC 2015-A0026, “Antibacterial activity of AMP (CSP-4).”

## SUPPLEMENTARY MATERIALS FIGURES AND TABLE


