# Supplementary material for: New antimicrobial peptide kills drug-resistant pathogens without detectable resistance
Source: Oncotarget. 2018 Feb 26;9(21):15616–34. doi: 10.18632/oncotarget.24582 (PMC5884652; doi:10.18632/oncotarget.24582)
Supplement: Supplementary file 1 [file oncotarget-09-15616-s001.pdf]

New antimicrobial peptide kills drug-resistant pathogens without detectable resistance

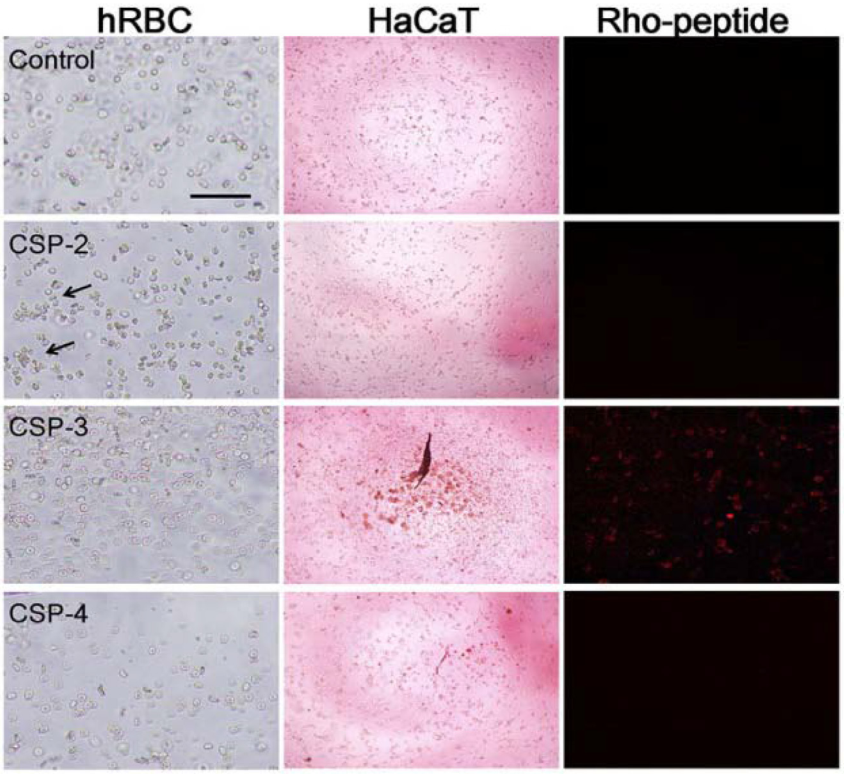

Supplementary Figure 1: The hemolytic activities of peptides were tested using hRBCs, and cytotoxicity was tested using a human keratinocyte line (HaCaT cells). The cells were treated with 400 µM peptide in PBS buffer containing 8% hRBCs (v/v). The samples were then incubated with mild agitation for 1 h at 37° C, and then spun down at 800 × *g* for 10 min. Changes in hRBC morphology were examined under a microscope. Cells were treated with 400 µM rhodamine-labeled peptides. HaCaT cells were seeded at 4 × 10^3 cells/well and then incubated for 24 h, treated with rhodamine-labeled peptide and incubated for an additional 24 h at 37° C in a humidified chamber under an atmosphere containing 5% CO_2. The cells were examined under an inverted fluorescence phase contrast microscope. Scale bar, 100 µm.

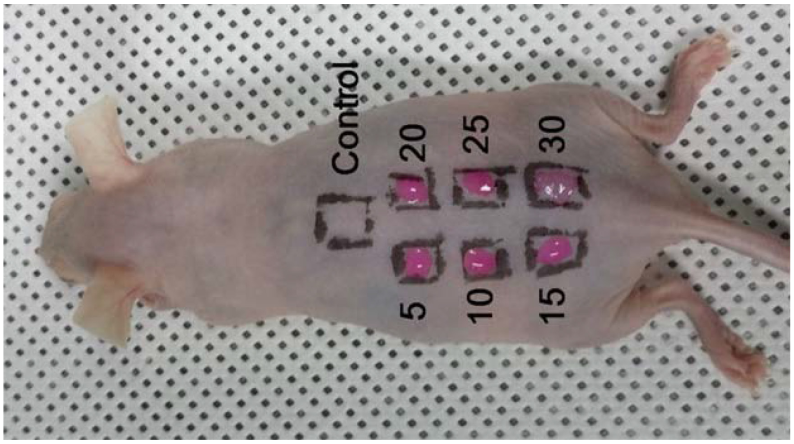

Supplementary Figure 2: Topical application of rhodamine-labeled CSP-4 to the skin of hairless mouse. Mice were anesthetized with isoflurane and immobilized. Rhodamine-labeled CSP-4 (200 µg/ml) was then applied to the skin in a topical spread. Diffusion of the peptide into the skin was assessed after 5, 10, 15, 20, 25 and 30 min.

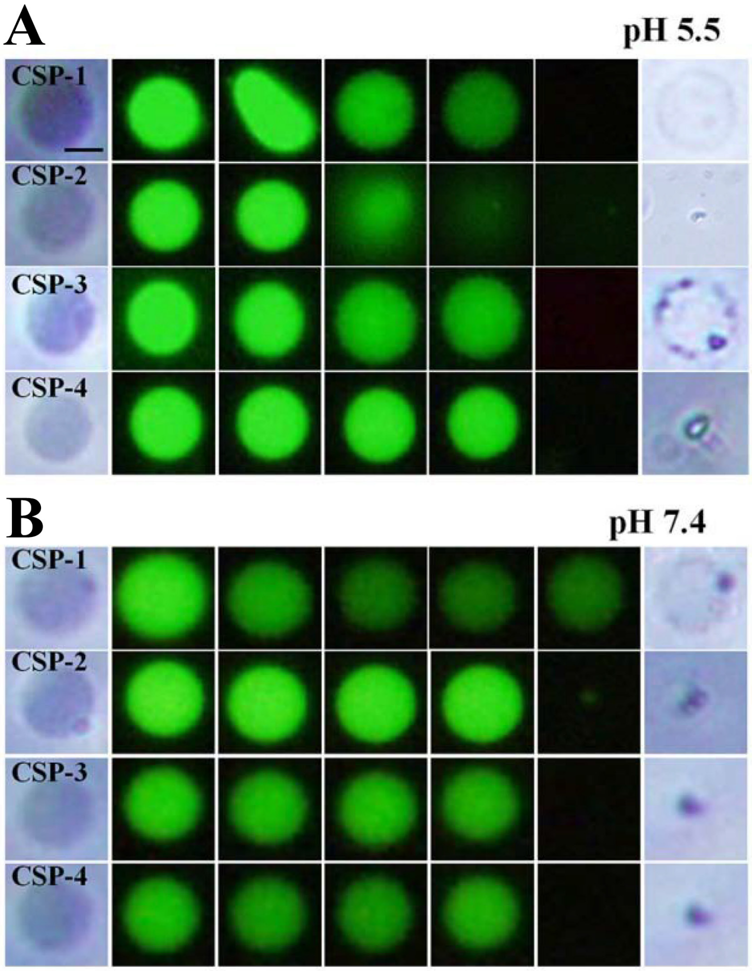

Supplementary Figure 3: Observation of GUVs (PE/PG) with peptides. GUVs settle due to the density difference between the sugar solution inside and outside the vesicles. GUVs were incubated with the indicated clavaspirin analogue (20 µM) in 5 mM HEPES buffer containing 0.1 M glucose at pH 5.5 and pH 7.4. Morphological changes and calcein leakage from the GUVs was monitored. GUVs were examined under an inverted fluorescence phase contrast microscope, and images were recoded using a digital CCD camera. Scale bar, 50 µm.

Supplementary Table 1: Sequence, molecular mass, retention time, and hemolytic and cytotoxic activities of clavaspirin and its analogues

Designation	Sequences	Molecular mass	Retention time (min)	HC_{10}/HC_{50}[a]	EC_{10}/EC_{50}[b]
Clavaspirin	FLRFIGSVIHGIGHLVHHIGVAL-NH_2	2492.01	35.2	37/64	66/247
CSP-1	FLRFAGSVIHGAGHLVHHIGVAL-NH_2	2407.84	26.6	40/334	120/>400
CSP-2	FLRF***K***GSVIHG***K***GHLVHHIGVAL-NH_2	2522.04	20.7	269/>400	>400/>400
CSP-3	F***K***RFIGSV***K***HGIGHLVHHIGVAL-NH_2	2450.96	21.4	>400/>400	84/188
CSP-4	FLRFIGSV***K***HG***K***GHLVHHIGVAL-NH_2	2450.96	20.2	>400/>400	>400/>400

The meanretention time was analyzed using C18 reversed phase-high-performance liquid chromatography (RP-HPLC). Samples were run using a linear gradient of 10–60 acetonitrile, containing 0.7% trifluoroacetic acid for 50 min.

[a]Hemolytic activity was determined in PBS buffer containing 8% hRBCs.

[b]Effective concentration in HaCaT cells was determined in DMEM buffer containing 10% FBS and 1% PS.
